# Supplementary material for: Gβγ engages PLCβ3 at multiple sites to reorient and facilitate its activation
Source: eLife. 2026 Sep 30;15:RP110382. doi: 10.7554/eLife.110382 (PMC13626650; doi:10.7554/eLife.110382)
Supplement: Supplementary file 2 [file elife-110382-supp1.docx]

**Supplementary File 1a.** Expression of HiBit-PLCβ3 variants as indicated by LgBit-complemented luminescence in intact cells.

| construct | *raw luminescence (photons)* | | | *n* |
| --- | --- | --- | --- | --- |
|  | mean | S.D. | *P* value^†^ |  |
| HiBit-PLCβ3 | 1.22E+07 | 6.27E+06 | - | 10 |
| R24A | 1.61E+07 | 6.51E+06 | 0.9768 | 3 |
| R24E | 3.25E+06 | 6.33E+05 | 0.1537 | 3 |
| L40E | 3.59E+06 | 1.26E+06 | 0.1905 | 3 |
| L40K | 2.62E+06 | 5.09E+05 | 0.1017 | 3 |
| D167A | 1.56E+07 | 4.73E+06 | 0.9928 | 3 |
| D167G | 1.27E+07 | 3.30E+06 | >0.9999 | 3 |
| R204A | 1.85E+07 | 4.91E+06 | 0.6269 | 3 |
| R204E | 3.27E+06 | 5.89E+05 | 0.156 | 3 |
| R24E+R204E | 3.51E+06 | 1.30E+06 | 0.1809 | 3 |
| R185E | 1.23E+07 | 8.58E+06 | >0.9999 | 4 |
| R215A | 1.19E+07 | 7.40E+06 | >0.9999 | 3 |
| R215E | 1.21E+07 | 4.90E+06 | >0.9999 | 3 |
| L222K | 1.37E+07 | 5.25E+06 | >0.9999 | 4 |
| F285E | 8.39E+06 | 4.37E+06 | 0.9484 | 4 |
| K183E | 1.67E+07 | 6.30E+06 | 0.8662 | 4 |
| HiBit-PLCβ3* | 6.46E+05 | 1.15E+05 | - | 5 |
| R199E* | 5.41E+05 | 1.20E+05 | 0.1928^‡^ | 5 |

^†^One-way ANOVAX, Dunnett’s multiple comparisons test vs. wild-type.

*Donor luminescence.

^‡^Welch’s t-test vs. wild-type.

**Supplementary File 1b.** Expression of HiBit-PLCβ3-CAAX variants as indicated by LgBit-complemented luminescence in intact cells.

| construct | *raw luminescence (photons)* | | | *n* |
| --- | --- | --- | --- | --- |
|  | mean | S.D. | *P* value^†^ |  |
| HiBit-PLCβ3-CAAX | 1.54E+08 | 1.67E+08 | - | 7 |
| R24A | 2.51E+07 | 1.32E+07 | 0.1474 | 4 |
| R24E | 3.49E+07 | 2.05E+07 | 0.2216 | 4 |
| L40E | 3.65E+07 | 2.28E+07 | 0.2356 | 4 |
| L40K | 2.61E+07 | 1.37E+07 | 0.1539 | 4 |
| D167A | 2.75E+07 | 1.48E+07 | 0.1632 | 4 |
| D167G | 3.03E+07 | 1.94E+07 | 0.1833 | 4 |
| R204A | 3.76E+07 | 2.40E+07 | 0.2456 | 4 |
| R204E | 4.46E+07 | 2.76E+07 | 0.319 | 4 |
| R24E+R204E | 2.98E+07 | 1.54E+07 | 0.1797 | 4 |
| R185E | 2.43E+08 | 1.59E+08 | 0.719 | 3 |
| R215A | 3.15E+07 | 2.04E+07 | 0.1925 | 4 |
| R215E | 3.48E+07 | 2.49E+07 | 0.2203 | 4 |
| L222K | 1.91E+08 | 1.29E+08 | 0.9998 | 3 |
| F285E | 2.00E+08 | 1.11E+08 | 0.9976 | 3 |
| K183E | 1.55E+08 | 8.66E+07 | >0.9999 | 3 |
| Hi-PLCβ3-CAAX* | 5.55E+05 | 1.32E+05 | - | 5 |
| R199E* | 5.37E+05 | 1.37E+05 | 0.8403^‡^ | 5 |

^†^One-way ANOVA, Dunnett’s multiple comparisons test vs. wild-type-CAAX.

*Donor luminescence.

^‡^Welch’s t-test vs. wild-type.

**Supplementary File 1c.** Angiotensin II-induced BRET between HiBit-PLCβ3-CAAX variants and Venus-Gβγ.

| construct | *ΔBRET* | | | *n* |
| --- | --- | --- | --- | --- |
|  | mean | S.D. | *P* value^†^ |  |
| HiBit-PLCβ3-CAAX | 0.01163 | 0.004365 | - | 10 |
| +GRK3ct | -0.000968 | 0.001502 | <0.0001 | 8 |
| R24A | 0.008984 | 0.001371 | 0.9249 | 5 |
| R24E | 0.004995 | 0.00116 | 0.0040 | 7 |
| L40E | 0.001863 | 0.00054 | <0.0001 | 5 |
| L40K | 0.002092 | 0.00071 | <0.0001 | 5 |
| D167A | 0.0306 | 0.01035 | <0.0001 | 5 |
| D167G | 0.01943 | 0.004322 | 0.0023 | 5 |
| R204A | 0.007136 | 0.001778 | 0.2798 | 5 |
| R204E | 0.003198 | 0.000906 | <0.0001 | 7 |
| R24E+R204E | 0.002536 | 0.001165 | 0.0008 | 4 |
| R185E | 0.000937 | 0.000516 | 0.0004 | 3 |
| R215A | 0.02478 | 0.00525 | <0.0001 | 5 |
| R215E | 0.02706 | 0.006269 | <0.0001 | 5 |
| L222K | 0.006882 | 0.00098 | 0.4898 | 3 |
| F285E | 0.002372 | 0.000248 | 0.0033 | 3 |
| K183E | 0.0116 | 0.002322 | >0.9999 | 4 |
| R199E | 0.003528 | 0.001421 | 0.0041 | 4 |

^†^One-way ANOVA, Dunnett’s multiple comparisons test vs. wild-type-CAAX.

**Supplementary File 1d.** Angiotensin II-induced PI(4,5)P2 hydrolysis mediated by HiBit-PLCβ3 variants.

| construct | *ΔBRET* | | | *n* |
| --- | --- | --- | --- | --- |
|  | mean | S.D. | *P* value^†^ |  |
| HiBit-PLCβ3 | 0.05237 | 0.005064 | - | 11 |
| +GRK3ct | 0.02145 | 0.003139 | <0.0001 | 11 |
| R24A | 0.053 | 0.008833 | >0.9999 | 3 |
| R24E | 0.04107 | 0.004471 | 0.0008 | 5 |
| L40E | 0.02582 | 0.004777 | <0.0001 | 5 |
| L40K | 0.02727 | 0.003389 | <0.0001 | 5 |
| D167A | 0.07282 | 0.001987 | <0.0001 | 3 |
| D167G | 0.06207 | 0.01019 | 0.0520 | 3 |
| R204A | 0.04634 | 0.002524 | 0.6015 | 3 |
| R204E | 0.03782 | 0.007242 | <0.0001 | 5 |
| R24E+R204E | 0.03578 | 0.0041 | <0.0001 | 4 |
| R185E | 0.01643 | 0.001868 | <0.0001 | 3 |
| R215A | 0.06183 | 0.005277 | 0.0636 | 3 |
| R215E | 0.06154 | 0.006815 | 0.0809 | 3 |
| L222K | 0.02474 | 0.001306 | <0.0001 | 3 |
| F285E | 0.01552 | 0.00182 | <0.0001 | 3 |
| K183E | 0.03727 | 0.009687 | 0.0002 | 3 |
| R199E | 0.02955 | 0.003595 | <0.0001 | 5 |

^†^One-way ANOVA, Dunnett’s multiple comparisons test vs. wild-type.

**Supplementary File 1e.** Angiotensin II-induced BRET between HiBit-PLCβ3 variants and Gα_q_-Venus.

| construct | *ΔBRET* | | | *n* |
| --- | --- | --- | --- | --- |
|  | mean | S.D. | *P* value^†^ |  |
| HiBit-PLCβ3 | 0.09643 | 0.02322 | - | 3 |
| R24A | 0.08169 | 0.01774 | 0.9344 | 3 |
| R24E | 0.09379 | 0.01895 | >0.9999 | 3 |
| L40E | 0.105 | 0.01828 | 0.9996 | 3 |
| L40K | 0.07575 | 0.008115 | 0.6243 | 3 |
| D167A | 0.1023 | 0.01475 | >0.9999 | 3 |
| D167G | 0.05385 | 0.01087 | 0.0103 | 3 |
| R204A | 0.0951 | 0.02177 | >0.9999 | 3 |
| R204E | 0.1066 | 0.01497 | 0.9973 | 3 |
| R24E+R204E | 0.1008 | 0.01792 | >0.9999 | 3 |
| R185E | 0.1094 | 0.02068 | 0.9753 | 3 |
| R215A | 0.1116 | 0.01287 | 0.9203 | 3 |
| R215E | 0.08856 | 0.01038 | 0.9999 | 3 |
| L222K | 0.08054 | 0.01358 | 0.8935 | 3 |
| F285E | 0.05337 | 0.001885 | 0.0092 | 3 |
| K183E | 0.1021 | 0.007104 | >0.9999 | 3 |
| R199E | 0.1259 | 0.01843 | 0.0620 | 5 |

^†^One-way ANOVA, Dunnett’s multiple comparisons test vs. wild-type.

**Supplementary File 1f.** Angiotensin II-induced BRET between HiBit-PLCβ3 variants and mem-Venus.

| Construct | *ΔBRET* | | | *n* |
| --- | --- | --- | --- | --- |
|  | mean | S.D. | *P* value^†^ |  |
| HiBit-PLCβ3 | 0.03869 | 0.005318 | - | 3 |
| R24A | 0.03012 | 0.00526 | 0.0619 | 3 |
| R24E | 0.03258 | 0.002824 | 0.3757 | 3 |
| L40E | 0.03697 | 0.003716 | >0.9999 | 3 |
| L40K | 0.03456 | 0.002387 | 0.8565 | 3 |
| D167A | 0.03445 | 0.003044 | 0.8352 | 3 |
| D167G | 0.02822 | 0.003124 | 0.0108 | 3 |
| R204A | 0.03021 | 0.006573 | 0.0667 | 3 |
| R204E | 0.03401 | 0.004711 | 0.7333 | 3 |
| R24E+R204E | 0.03131 | 0.003755 | 0.1608 | 3 |
| R185E | 0.02671 | 0.002865 | 0.0023 | 3 |
| R215A | 0.0381 | 0.004039 | >0.9999 | 3 |
| R215E | 0.03365 | 0.005771 | 0.6405 | 3 |
| L222K | 0.03058 | 0.003968 | 0.0911 | 3 |
| F285E | 0.02902 | 0.002389 | 0.0232 | 3 |
| K183E | 0.02648 | 0.002579 | 0.0018 | 3 |
| R199E | 0.03633 | 0.003283 | 0.9927 | 5 |

^†^One-way ANOVA, Dunnett’s multiple comparisons test vs. wild-type.

**Supplementary File 1g.** Angiotensin II-induced PI(4,5)P2 hydrolysis mediated by HiBit-PLCβ3-CAAX variants.

| construct | *ΔBRET (normalized to wt PLCβ3-CAAX)* | | | *n* |
| --- | --- | --- | --- | --- |
|  | mean | S.D. | *P* value^†^ |  |
| +GRKct RQ | 1.005 | 0.1263 | - | 7 |
| +GRKct | 0.306 | 0.06239 | ` | 7 |
| L40E | 0.5392 | 0.1517 | <0.0001 | 7 |
| R185E | 0.2425 | 0.04493 | <0.0001 | 7 |
| K183E | 0.47 | 0.09543 | <0.0001 | 7 |

^†^One-way ANOVA, Dunnett’s multiple comparisons test vs. wild-type.
