# Supplementary material for: Gβγ engages PLCβ3 at multiple sites to reorient and facilitate its activation
Source: eLife. 2026 Sep 30;15:RP110382. doi: 10.7554/eLife.110382 (PMC13626650; doi:10.7554/eLife.110382)
Supplement: Peer review history

# Editor report: eLife Assessment

**DOI:** 10.7554/eLife.110382.3.sa0

**Authors:** John Janetzko

**Affiliations:** University of Colorado Anschutz Medical Campus United States

**Keywords:** Convincing, Important

This **important** study combines cryo-EM, biochemical, and cell-based assays to examine how Gβγ interacts with and potentiates PLCβ3. The authors present evidence for multiple Gβγ interaction surfaces and argue that Gβγ primarily enhances PLCβ3 activity after membrane recruitment rather than serving mainly as a membrane-recruitment factor. Following additional experimental support, the evidence in support of their conclusions is **convincing**.

# Referee report: Reviewer #1 (Public review)

**DOI:** 10.7554/eLife.110382.3.sa1

The manuscript by Fisher et al describes the molecular mechanism underlying how G beta gamma subunits engage with the beta 3 isoform of PLC. The paper used a combination of cryo EM, BRET assays, and biochemical assays of PLC beta activity. A key discovery is that G beta gamma is not sufficient to drive membrane binding by itself and instead promotes G alpha activation. The work is important, but suffers slightly from some ambiguity in the actual interface that is present in their cryo EM model, as crosslinkers could stabilise a transient and non-native complex. This is somewhat abrogated by the careful mutational analysis, which shows that mutation of any of these three sites does somewhat block PLC beta G beta gamma activation. However, there could be some improvement in the presentation of this data, as well as possible mutant selection. Overall, this paper is a nice complement to the Falzone et al paper showing the membrane bound complex of PLCB3 on membranes, with this work building on this work, highlighting the importance this will have in our full understanding of PLC beta activation.

Major concerns

My most major concern is the potential that this interface is artefactual based on the crosslinking strategy utilised. Here are thoughts on how this could be better validated, presented in a more convincing way.

(1) The authors main claim is that there is a degree of plasticity of G beta gamma binding to the PLC beta 3 isoform, with three possible binding sites. The main complication of this is of course the possibility that the crosslinking stabilises a non-native complex, driven by a mutated cysteine.

Because of this any other additional details about this interface are going to be critical for the scientific audience to judge if this is accurate.

What would greatly help figure 1, is an evolutionarily conservation analysis of the novel Gbg interface in PLC, to see how well this is conserved, and compare this to the conservation of the previously annotated sites. Conservation of these sites on both the G beta gamma and PLC side would help justify this as a native complex.

This also will help orient the reader to the identity of the mutated residues assayed in figure 3.

(2) The g beta gamma orientation is also different than what I have observed in previous g beta gamma effector structures. Is there any precedent for this as an effector interface? A supplemental figure comparing this structure to other g beta gamma interfaces from other enzymes, for example recent tesmer structure with PI3K.

(3) The mutational analysis in Figure 2D-G seems to give some strange results, and I have some question why certain residues were chosen rather than others. Mutation of the Gbg side will be more complicated as of course that can effect any of the three surfaces. My main question is that from the way fig 2A is oriented that the main salt bridge in their novel interface to me looks like R199-D228, with K183 being in the wrong orientation to E226, and D167 being far from any charged residues. Why did the authors not make the corresponding R199 to D or E mutation?

(4) To help reader interpretation of Figure 2A, I would recommend a supplemental figure showing the density for interfacial residues, as that also would increase confidence in the interface.

Comment on revised version.

After revision the authors have addressed all of my concerns.

# Referee report: Reviewer #2 (Public review)

**DOI:** 10.7554/eLife.110382.3.sa2

In this manuscript, the authors dissect how Gβγ potentiates PLCβ3 signaling in cells. Using engineered crosslinking to stabilize a Gβγ-PLCβ3 complex, single particle cryo-EM, and cell-based functional assays, they identify map multiple putative Gβγ interaction surfaces on PLCβ3, including a previously unrecognized binding mode. Structure-guided mutagenesis supports the functional relevance of these interactions and suggests that Gβγ potentiation is not primarily mediated by PLCβ3 membrane recruitment, but instead enhances PLCβ3 activity after the lipase is already at the membrane.

Previous reconstitution work on membrane surface (Falzone & MacKinnon, 2023) proposed a recruitment/partitioning-centric model in which Gβγ increases PLCβ3 output largely by elevating its membrane surface concentration, whereas Gαq primarily increases catalytic turnover; under those reconstitution conditions, the two inputs can combine approximately multiplicatively. In receptor-driven cellular signaling, however, PLCβ3 is robustly recruited to the plasma membrane upon Gαq activation, which raises the question of whether Gβγ contributes mainly through additional recruitment or through a post-recruitment mechanism once PLCβ3 is already at the membrane.

This manuscript helps address that gap by using membrane-anchored PLCβ3 and complementary cellular readouts to separate "getting PLCβ3 to the membrane" from "boosting activity once PLCβ3 is already there." Their results argue that, in cells, membrane recruitment is largely dominated by Gαq·GTP, while Gβγ can further potentiate PIP2 hydrolysis after membrane association, consistent with a modulatory role at the membrane rather than primary recruitment.

Overall, the work provides a structural and mechanistic framework for Gβγ-PLCβ3 cooperation and helps clarify the basis of Gq pathway amplification.

Comments on revised version.

The authors have reasonably addressed my comments.

# Referee report: Reviewer #3 (Public review)

**DOI:** 10.7554/eLife.110382.3.sa3

Summary:

PLCβ3 is activated by both Gαq and Gβγ subunits. This paper follows previous solution and cryoEM studies of the PLCβ3 / Gβγ complex to delineate the molecular details of activation using cellular BRET assays and cryoEM.

Strengths:

The authors find evidence for multiple binding sites on PLCβ3 for Gβγ and suggest that Gβγ is not bone fide activator per se but enhances Gαq activation by positioning the catalytic site towards substrate. The authors also find that this activation is not through recruitment of the enzyme to the membrane by Gβγ released upon G protein activation in accord with other PLCβ enzymes.

Weaknesses:

(1) The main issue is that the author's mechanism does not fully explain how Gβγ activation occurs for PLCβ2 in reconstituted systems in the absence of Gαq subunits but will be investigating this in future studies.

# Author comment: Author response

**DOI:** 10.7554/eLife.110382.3.sa4

**Authors:** Isaac J Fisher, Kanishka Senarath, Kennedy Outlaw, Kaushik Muralidharan, Elisabeth E Garland-Kuntz, Michelle M Van Camp, Thomas Komay, Leon F Laskowski, Asuka Inoue, Evi Kostenis, Nevin A Lambert, Angeline Lyon

**Affiliations:** Purdue University West Lafayette West Lafayette United States; Augusta University Augusta United States; Stanford University Palo Alto United States; Center for Clinical and Translational Research, Abigail Wexner Research Institute at Nationwide Children's Columbus United States; Purdue University West Lafayette West Lafayette United States; Purdue University West Lafayette West Lafayette United States; Purdue University West Lafayette West Lafayette United States; Purdue University West Lafayette West Lafayette United States; Kyoto University Kyoto Japan; University of Bonn Bonn Germany; Augusta University Augusta United States; Purdue University West Lafayette West Lafayette United States

The following is the authors’ response to the original reviews.

**Public Reviews:**

**Reviewer #1 (Public review):**

The manuscript by Fisher et al describes the molecular mechanism underlying how G beta gamma subunits engage with the beta 3 isoform of PLC. The paper used a combination of cryo EM, BRET assays, and biochemical assays of PLC beta activity. A key discovery is that G beta gamma is not sufficient to drive membrane binding by itself, and instead promotes G alpha activation. The work is important, but suffers slightly from some ambiguity in the actual interface that is present in their cryo EM model, as crosslinkers could stabilise a transient and non-native complex. This is somewhat abrogated by the careful mutational analysis, which shows that mutation of any of these three sites does somewhat block PLC beta G beta gamma activation. However, there could be some improvement in the presentation of this data, as well as possible mutant selection. Overall, this paper is a nice complement to the Falzone et al paper, showing the membrane-bound complex of PLCB3 on membranes, with this work building on this work, highlighting the importance this will have in our full understanding of PLC beta activation.

Thank you for the positive feedback.

Major concerns:

My biggest concern is the potential that this interface is artefactual based on the crosslinking strategy utilised. Here are thoughts on how this could be better validated, presented in a more convincing way.

(1) The authors' main claim is that there is a degree of plasticity of G beta gamma binding to the PLC beta 3 isoform, with three possible binding sites. The main complication of this is, of course, the possibility that the crosslinking stabilises a non-native complex, driven by a mutated cysteine.

Because of this, any other additional details about this interface are going to be critical for the scientific audience to judge if this is accurate.

What would greatly help Figure 1 is an evolutionary conservation analysis of the novel Gbg interface in PLC, to see how well this is conserved, and compare this to the conservation of the previously annotated sites. Conservation of these sites on both the G beta gamma and PLC side would help justify this as a native complex.

This will also help orient the reader to the identity of the mutated residues assayed in Figure 3.

We agree that crosslinking can capture non-physiologically relevant interfaces. However, because we do not observe any crosslinking between Gβγ and a PLCβ3 variant that retains a cysteine in the X–Y linker or between PLCβ3 and any other cysteine in the Gβγ heterodimer, we believe it is site-specific.

The question about sequence conservation in the Gβγ–PLCb3 interfaces is interesting and we have included this information in Figures S8 and S9.

(2) The g beta gamma orientation is also different than what I have observed in previous g beta gamma effector structures. Is there any precedent for this as an effector interface? A supplemental figure comparing this structure to other g beta gamma interfaces from other enzymes, for example recent Tesmer structure with PI3K.

We agree that the orientation of Gβ in the crosslinked structure is different. We include a comparison of this reconstruction to other published Gβγ–effector complexes as Figure S6.

(3) The mutational analysis in Figure 2D-G seems to give some strange results, and I have some question why certain residues were chosen rather than others. Mutation of the Gbg side will be more complicated, as of course that can affect any of the three surfaces. My main question is that, from the way Figure 2A is oriented, the main salt bridge in their novel interface to me looks like R199-D228, with K183 being in the wrong orientation to E226, and D167 being far from any charged residues. Why did the authors not make the corresponding R199 to D or E mutation?

Thank you for pointing this out, and we expanded our analysis to include this residue. The R199A and R199E mutations had no defects in basal or Ga_q_-stimulated activities. However, R199A had 3-fold lower activation by Gβγ, while R199E was not activated in this assay. The R199E mutation also had significantly decreased agonist-dependent BRET with Gβγ and decreased PI(4,5)P2 hydrolysis, while retaining robust recruitment to the plasma membrane by Gα_q_. These data is included in the main text and Figures 2-4.

(4) To help the reader's interpretation of Figure 2A, I would recommend a supplemental figure showing the density for interfacial residues, as that also would increase confidence in the interface.

Thank for the suggestion. In revised Figure S3, we show the Gβγ–PLCb3 D892-PH_cys_ complexes determined in this study at different contour levels.

**Reviewer #2 (Public review):**

In this manuscript, the authors dissect how Gβγ potentiates PLCβ3 signaling in cells. Using engineered crosslinking to stabilize a Gβγ-PLCβ3 complex, single particle cryo-EM, and cell-based functional assays, they identify and map multiple putative Gβγ interaction surfaces on PLCβ3, including a previously unrecognized binding mode. Structure-guided mutagenesis supports the functional relevance of these interactions and suggests that Gβγ potentiation is not primarily mediated by PLCβ3 membrane recruitment, but instead enhances PLCβ3 activity after the lipase is already at the membrane.

Previous reconstitution work on the membrane surface (Falzone & MacKinnon, 2023) proposed a recruitment/partitioning-centric model in which Gβγ increases PLCβ3 output largely by elevating its membrane surface concentration, whereas Gαq primarily increases catalytic turnover; under those reconstitution conditions, the two inputs can combine approximately multiplicatively. In receptor-driven cellular signaling, however, PLCβ3 is robustly recruited to the plasma membrane upon Gαq activation, which raises the question of whether Gβγ contributes mainly through additional recruitment or through a post-recruitment mechanism once PLCβ3 is already at the membrane.

This manuscript helps address that gap by using membrane-anchored PLCβ3 and complementary cellular readouts to separate "getting PLCβ3 to the membrane" from "boosting activity once PLCβ3 is already there." Their results argue that, in cells, membrane recruitment is largely dominated by Gαq·GTP, while Gβγ can further potentiate PIP2 hydrolysis after membrane association, consistent with a modulatory role at the membrane rather than primary recruitment.

Overall, the work provides a structural and mechanistic framework for Gβγ-PLCβ3 cooperation and helps clarify the basis of Gq pathway amplification. The manuscript is generally strong, but some issues need to be addressed.

Thank you for the positive comments.

Major comments:

(1) BMOE/BM(PEG)2 crosslinking may enforce a non-native docking geometry, potentially compromising the physiological relevance and precision of the Gβγ-PLCβ3 interface as described. Although a >50% 1:1 crosslinked complex is formed and remains active, the solution maps show lower local resolution for Gβγ, consistent with a dynamic, potentially heterogeneous, interface. One interface is captured via a single engineered cysteine pair (PLCβ3 E60C-Gβ C271), which could potentially bias the pose. It would be helpful if the authors could provide additional orthogonal support (e.g., alternative crosslinked sites) and bolster the clarification of its uniqueness and relevance.

We did attempt to isolate other crosslinked complexes. PLCβ3-D892 self-crosslinked under all reaction conditions, while PLCβ3-D892 XY_Cys_, which retains an endogenous cysteine within the X–Y linker (C516), did not result in any crosslinked product when incubated with Gβγ. Only the PLCβ3-D892 E60C crosslinked to Gβγ. With the exception the C68S mutation at the C-terminus of Gg to eliminate its prenylation site, all endogenous cysteines were retained in both Gβ and Gγ. Indeed, Gβ contains two solvent-exposed cysteines in its canonical effector binding surface (C204 and C271), but we did not observe any crosslinker density involving C204. While we cannot exclude the possibility that crosslinking occurred between PLCβ3-D892 E60C and other residues in Gβγ, we were unable to identify any 2D classes corresponding to these alternative conformations. These observations, together with the high efficiency of crosslinking, are consistent with a stable and persistent interaction.

(2) In the crosslinked structure, the authors report that GβD228 interacts with PLCβ3 R199 and K183. In Figure 2A, R199 appears closer to Gβ D228 than K183, yet only K183 is functionally tested. Testing R199 (e.g., R199E/R199A) would strengthen the structure-guided validation of this interface.

We agree, and functional analysis of PLCb3 R199E is included in the revised manuscript (see Figures 2-4).

(3) The mutagenesis strategy appears inconsistent across figures/assays, which makes it difficult to interpret phenotypes and directly link the functional data to the proposed interfaces. For example, in Figure 2E, we see R185L but R215E, while residue L40 is mutated to Gly in the IP accumulation assays but to Glu/Lys (L40E/K) in the BRET assays (Figures 3B/3D/3F). The authors should (i) clearly justify the rationale for each substitution (conservative vs charge-reversal, interface disruption, etc.) and (ii), where possible, test the same mutants across assays (or provide evidence that alternative substitutions yield consistent conclusions).

Mutagenesis experiments were initially carried out independently in the Lambert and Lyon Labs. As the study progressed, additional mutants were identified and/or designed based on results from both groups. The residues subject to mutagenesis are overall consistent across the different assays, with differences in the identity of the mutation varying in some cases. The L40G mutation is one such example, where given its modest impact on Gβγ-mediated activation in the IP accumulation assay, more impactful changes were made (L40E and L40K) for the BRET and signaling assays. In the revision, we now state that mutations were designed to maximally disrupt the three observed interfaces, such as by changing the size of the side chain and/or introducing charge reversal mutants.

**Reviewer #3 (Public review):**

Summary:

PLCβ3 is activated by both Gαq and Gβγ subunits. This paper follows previous solutions and cryoEM studies of PLCβ3 / Gβγ, trying to understand the molecular details of activation using cellular BRET assays and cryoEM.

Strengths:

The authors find evidence for multiple binding sites on PLCβ3 for Gβγ and suggest that Gβγ is not bone fide activator per se but enhances Gαq activation by positioning the catalytic site towards substrate, although this is not completely convincing. Although these sites may not naturally be operative, the authors might want to develop the potential role of these sites.

The authors also find that this activation is not through recruitment of the enzyme to the membrane by Gβγ released upon G protein activation, in accord with other PLCβ enzymes, but not for PLCβ3, and again, the authors might want to develop this point further.

Thank you for the suggestions. We are investigating whether the other PLCb isoforms contain multiple Gβγ binding sites and the relative importance of preactivation by Ga_q_ for a manuscript in preparation.

Weaknesses:

(1) I'm confused as to why the authors feel that their mechanism is distinct from the two-state enzyme, the synergistic activation proposed by Ross in 2011, using a primarily thermodynamic argument. As written, the authors appear to be very reliant on structural and BRET studies that do not give the details that would disprove this interpretation. The main issue is that the author's mechanism does not fully explain how Gβγ activation occurs for PLCβ2 in reconstituted systems in the absence of Gαq subunits.

The reconstitution experiments are under extremely artificial conditions, using nM-µM of purified proteins and liposomes that contain up to 30% PI(4,5)P2. Under these conditions, we think the increased activity is due to interfacial activation promoted by Gβγ binding to the lipase once it is associated with the liposome surface. This would be sufficient to account for the dose-dependent increase in both PLCb2 and PLCb3 activity as a function of Gβγ concentration. Given the higher basal activity of PLCβ2 and its decreased sensitivity to activation by Ga_q_, one possible explanation is that this isoform differs in its autoinhibition and/or structure of its proximal CTD that Ha2’ displacement is not a prerequisite for activation. In addition, Gβγ may also be a direct activator of PLCβ2. Further studies, ideally in cell-based systems, are needed to answer these questions.

(2) In a recent study, McKinnon presents a model showing that Gαq and Gβγ activate PLCβ3 by two distinct pathways and that activation by Gβγ occurs through membrane recruitment. It is not surprising that the authors find that this is not true since the pelleting method used by McKinnon is subject to error. The authors should directly address the limitations of this previous work and the changes in proteoliposomes with sedimentation that alter partition coefficients. Although the inability of Gβγ to drive membrane binding is in accord with the quantitative studies of Scarlata, showing that the affinity of PLCβ3 to Gβγ is fairly weak as compared to the intrinsic membrane partition coefficient.

We have added some of the limitations of proteoliposome sedimentation experiments to the discussion.

(3) It was proposed many years ago that in signaling complexes Gαq - Gβγ may not have to fully dissociate when binding PLCβ, but rather shift their relative orientation when binding to PLCβ to allow activation. Is their model consistent with this? Is it possible that PLCβ3 keeps Gβγ from diffusing to enhance the rate of Gq / Gβγ re-association?

Our crosslinked complex is compatible with simultaneous binding of a Gα_q_-Gβγ heterotrimer to the PLCb3, without disrupting the observed interface. If Gαq were to interact with the Gβγ molecules bound to the PH or EF hands, the interaction would be mediated by the N-terminal helix of Gα_q_. It is possible Gβγ–PLCβ3 interactions may slow heterotrimer reassociation, but this may be complicated by the intrinsic GAP activity of the lipase.

(4) The authors find that Gβγ binds multiple sites, and it is clear that the PH domain site is the primary one in accord with previous work. Could these weaker sites be an artifact of the elevated concentrations used in cryoEM and BRET assays?

While more studies have focused on the PH domain as a Gβγ binding site, our data does confirm the EF hands are also functionally relevant. To our knowledge, the role of the EF hands has not been investigated in this capacity until very recently, and so we hesitate to label them primary or secondary. It is possible the EF hands may be a lower-affinity site for Gβγ and the protein concentrations needed in cryo-EM drive complex formation. However, it is also possible the concentration of free Gβγ adjacent to an activated receptor may be high enough to saturate the PH and EF hand binding sites.

(5) Although their assays infer differences in binding affinities, it would strengthen the paper if the authors could estimate the association energies of these different binding sites. This estimation would also address the concern stated above.

We appreciate this suggestion and quantifying the affinities of the Gβγ–PLCβ3 interactions is the subject of future studies.

**Recommendations for the authors:**

**Reviewer #1 (Recommendations for the authors):**

Please correct PIP2 to the correct PIP2 (many examples throughout).

These have been corrected.

**Reviewer #2 (Recommendations for the authors):**

Minor comments:

(1) Figure S1B: The lane-condition labels above the third gel appear to be incorrect, as both lanes are marked identically (Gβγ +/+, PLCβ variant +/+, BMOE +/+) despite clearly different banding patterns. Please confirm.

We have confirmed the markings above the gels are correct.

(2) In Figure S2, the authors show three fitted models, but the helical density for Gβγ cannot be seen in two of them at the displayed contour level. The authors should provide views of the map at different contour levels (thresholds) to better support the model fitting. Otherwise, it is difficult to assess whether the Gβγ subunit could adopt alternative orientations (i.e., whether it may be rotated) within the density.

We have included a new figure (Figure S3) that provides images of the maps at different contour levels.

(3) Page 5: "where PLCβ3 is increased by the overexpression of either Gβγ or Gαq" should be revised to "where PLCβ3 activity is ...".

This sentence has been corrected.

(4) Figure 2: Please label residue R215 in Figure 2A/2B (or the relevant structural panel), since R215E is tested in 2E but the position is not shown.

R215 is now included in Figure 2C.

(5) Page 19, Figure 2 legend: "Changes ... Figure S3" should be "Changes ... Figure S5".

We have corrected this figure call.

**Reviewer #3 (Recommendations for the authors):**

The studies seem well carried out, although more details regarding the BRET controls and the significance of the values should be included.

We have revised the captions to provide more details about the experimental controls and a brief description of significance. Individual p-values are included in the supplemental tables.
